# Noradrenaline, Serotonin, GABA, and Glycine in Cerebrospinal Fluid during Labor Pain: A Cross-Sectional Prospective Study

**DOI:** 10.1155/2017/2752658

**Published:** 2017-06-18

**Authors:** Pornpan Chalermkitpanit, Atikun Thonnagith, Phatthanaphol Engsusophon, Somrat Charuluxananan, Sittisak Honsawek

**Affiliations:** ^1^Department of Anesthesiology, Faculty of Medicine, Chulalongkorn University, King Chulalongkorn Memorial Hospital, Thai Red Cross Society, Bangkok, Thailand; ^2^Department of Biochemistry, Faculty of Medicine, Chulalongkorn University, King Chulalongkorn Memorial Hospital, Thai Red Cross Society, Bangkok, Thailand

## Abstract

**Background and Aims:**

The inhibitory pathways that play a role in spinal modulation include local interneurons and descending control. Clinical data regarding the role of these pathways in acute pain is lacking. Accordingly, the aim of this study was to evaluate cerebrospinal fluid (CSF) levels of noradrenaline, serotonin, gamma-aminobutyric acid (GABA), and glycine in parturients with labor pain compared to those without labor pain.

**Methods:**

One hundred term uncomplicated pregnant women receiving spinal anesthesia for cesarean section were enrolled in this prospective cross-sectional study. CSF noradrenaline, serotonin, GABA, and glycine levels were analyzed by enzyme-linked immunosorbent assay. Labor pain score was assessed by numerical rating scale.

**Results:**

Median CSF serotonin concentration in parturients with labor pain was significantly lower than in those without pain (*p* < 0.001). Median CSF glycine level in the labor pain group was significantly higher than in the control group (*p* < 0.001). There were no significant differences in median CSF level of noradrenaline or GABA between parturients with and without labor pain. Subsequent analysis showed labor pain scores to be negatively correlated with CSF serotonin (*r* = −0.217, *p* = 0.04) but positively correlated with CSF glycine (*r* = 0.415, *p* < 0.001).

**Conclusion:**

CSF serotonin and glycine were significantly correlated with labor pain scores. These findings suggest that the serotonergic and glycinergic systems may play a role in spinal modulation of visceral pain.

## 1. Introduction

Noxious stimuli from the periphery are modulated in the dorsal horn of the spinal cord before signals are transmitted to the thalamus and the cortex, and then the perception of pain develops. The two major pathways that attenuate nociceptive input in the dorsal horn consist of local control from inhibitory interneurons and the descending inhibitory system from the supraspinal level. According to the gate control theory, interneurons in the dorsal horn release gamma-aminobutyric acid (GABA) and glycine, which mediate inhibitory postsynaptic potential to reduce nociceptive impulse [1, 2]. The descending inhibitory system, which consists of the periaqueductal gray, the rostral ventromedial medulla, and the locus coeruleus in the brain stem, releases serotonin, noradrenaline, and endorphin to modulate pain at the dorsal horn [3]. Moreover, findings from animal studies have revealed interrelationship between the descending inhibitory system and inhibitory interneurons relative to nociceptive modulation in the spinal cord [4–7].

Study in patients with posttraumatic pain found significantly decreased cerebrospinal fluid (CSF) concentration of noradrenaline and significantly increased CSF concentration of serotonin [8]. In pregnant women, CSF noradrenaline concentration was increased in parturients with labor pain, as compared to those without labor pain [9]. Recently, Buvanendran et al. reported decreased CSF noradrenaline concentration during the early postoperative period after total knee replacement [10]. The question that then logically follows is, are the descending noradrenergic and serotonergic systems inhibited or activated during acute pain? Based on our review of the literature, there has been limited clinical study regarding the role of inhibitory interneurons in an acute pain setting. A recent study by Bonin et al. reported that GABA interneurons in the spinal dorsal horn generated tonic inhibitory conduction to regulate acute nociceptive input in mice [11]. Sethuraman et al. found CSF concentrations of GABA and glycine in pregnant women with labor pain to be significantly higher than in pregnant women without labor pain [12]. In contrast, CSF GABA concentration was significantly reduced in pregnant women with labor pain and was strongly related to increased incidence of postpartum depression [13]. Given the limited and sometimes conflicting scientific evidence relating to the role of inhibitory neurotransmitters in pain modulation, no conclusive determination about this relationship has yet been established. Labor pain was reported as being an excellent model of acute pain [14].

The aim of this study was to evaluate cerebrospinal fluid (CSF) levels of noradrenaline, serotonin, gamma-aminobutyric acid (GABA), and glycine in parturients with labor pain compared to those without labor pain in order to investigate the role of the descending inhibitory system and inhibitory interneurons in the spinal cord in an acute pain setting.

## 2. Materials and Methods

### 2.1. Ethical Aspect

This study complied with the guidelines set forth in the Declaration of Helsinki and all of its subsequent amendments. The protocol for this study was approved by the Institutional Review Board of the Faculty of Medicine, Chulalongkorn University, Bangkok, Thailand (IRB number 369/56).

### 2.2. Study Population

One hundred women with term, uncomplicated pregnancy undergoing cesarean section under spinal anesthesia were included in this prospective cross-sectional study. Written informed consent was obtained from all enrolled participants. Women with complicated pregnancy (e.g., eclampsia, placental abruption, uterine abruption, or fetal distress) or who had contraindication for use of spinal anesthesia (e.g., coagulopathy, aortic stenosis, or infection at the blocking area) were excluded. Women with chronic pain, neuropathic pain, neurological disease, or history of recent use of substances that act on the central nervous system were also excluded.

Parturients were divided into 2 groups. Pregnant women scheduled for elective cesarean section reporting no labor pain were allocated into the control group (*n* = 50). Pregnant women undergoing emergency cesarean section with labor pain score of at least 5 out of 10 as measured by verbal numerical rating scale (NRS 0–10) for a duration longer than 2 hours were allocated into the labor pain group (*n* = 50). Verbal numerical rating scale was previously validated for assessment of acute pain [15]. Average pain score of the last three uterine contractions, duration of labor pain from the time point when NRS was greater than 5, and pain medication received were recorded.

Pregnant women in both study groups received standard anesthetic care and monitoring during surgery. A 25-gauge or 27-gauge Quincke spinal needle was inserted into the subarachnoid space via the L2-3 or L3-4 interspace. Patients from whom blood-stained CSF was extracted were excluded from the study. The first few drops of CSF were discarded to reduce blood contamination before collecting 1.5 mL of CSF into an ethylene diamine tetra-acetic acid (EDTA) tube. Bupivacaine and morphine were then injected into the intrathecal space to provide spinal anesthesia for surgery. CSF samples were frozen at −20°C for subsequent assay analysis. The primary outcome was CSF concentrations of noradrenaline, serotonin, GABA, and glycine between pregnant women with labor pain and those without labor pain. The secondary outcomes were correlations between each neurotransmitter and pain intensity and pain duration in the labor pain group and correlations among the 4 neurotransmitters.

### 2.3. Laboratory Methods

Double-blind quantitative determination of noradrenaline, serotonin, GABA, and glycine concentrations in cerebrospinal fluid was performed using commercially available sandwich enzyme-linked immunosorbent assay (ELISA) kits, according to manufacturer's protocol. Noradrenaline (KA1891) and serotonin (KA1894) ELISA kits were obtained from Abnova Corporation, Taipei, Taiwan. The detection limits for noradrenaline and serotonin were 50 ng/L and 5 *µ*g/L, respectively. GABA (K7012) and glycine (K7013) ELISA kits were obtained from Immundiagnostik AG, Bensheim, Germany. The detection limits for GABA and glycine were 0.024 *µ*mol/L and 14.3 *µ*mol/L, respectively.

### 2.4. Statistical Analysis

A power analysis was performed using CSF glycine values in parturients with labor pain as the primary outcome measure [12]. The sample size was calculated so that a mean difference of 10% between groups would permit a type 1 error probability of *α* = 0.05 (two-tailed test) with altered CSF glycine values in the labor pain group, and the null hypothesis would be retained with *β* = 0.10. Therefore, a sample size of 43 patients per group was required in each group. Continuous data are expressed as mean ± standard deviation (SD), and categorical data are shown as frequency and percentile. All results were tested for normal distribution using Shapiro-Wilk test. Nonparametric data are presented as median and interquartile range (IQR, 25th and 75th percentiles). Differences in neurotransmitter concentrations were examined using Mann–Whitney* U* test. Correlations between neurotransmitter levels and pain intensity/pain duration and correlations among the 4 evaluated neurotransmitters were analyzed using Spearman's correlation coefficient. All statistical analyses were performed using SPSS Statistics version 22.0 (SPSS, Inc., Chicago, IL, USA). A *p* value < 0.05 was regarded as being statistically significant.

## 3. Results

### 3.1. Demographic and Clinical Characteristics

One hundred pregnant women were prospectively enrolled in this study. Seven women in the control group and 5 women in the labor pain group were excluded due to blood-stained CSF. As such, data from 43 women in the control group and 45 women in the labor pain group were included in the final analysis. Demographic, anthropometric, and clinical characteristics of pregnant women in both groups are shown in Table 1. There were no statistically significant differences in weight, height, body mass index, or gestational age between groups. However, mean age and indication for cesarean section were found to be significantly different between groups (Table 1). Regarding indication for cesarean section, most women in the control group had previous cesarean section, while most women in the labor pain group had cephalopelvic disproportion (CPD). Women in the labor pain group reported severe preoperative pain [numerical rating score (NRS) of 9 (IQR 7–10)], with a median pain duration of 325 (IQR 240–525) minutes. Pregnant women in the control group reported no pain (NRS = 0).

### 3.2. CSF Neurotransmitters in Parturients with and without Labor Pain

**Figure 1:**
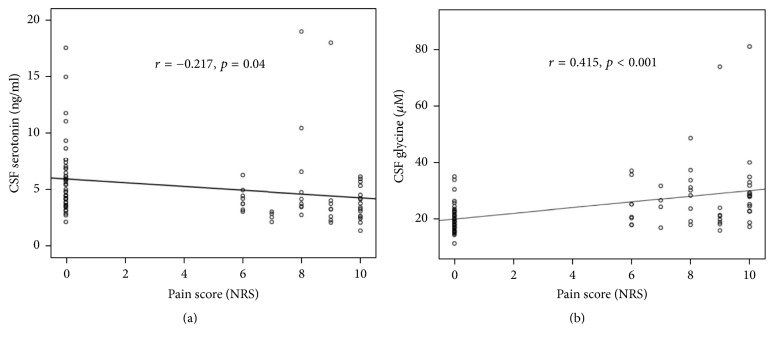
Correlation between pain scores and cerebrospinal fluid (CSF) levels of serotonin and glycine as measured by numerical rating scale (NRS): (a) CSF serotonin levels (Spearman's coefficient: *r* = −0.217; *p* = 0.04); (b) CSF glycine levels (Spearman's coefficient: *r* = 0.415; *p* < 0.001).

Concentrations of CSF neurotransmitters in pregnant women in the control and labor pain groups are shown in Table 2. CSF serotonin concentration in women with labor pain was significantly lower than in those without labor pain (*p* < 0.001). The level of glycine in the labor pain group was significantly higher than the glycine level in the control group (*p* < 0.001). There were no significant differences in CSF concentrations of noradrenaline or GABA between groups. Subsequent analysis revealed labor pain scores to be negatively correlated with CSF serotonin concentration (*r* = −0.217, *p* = 0.04) and positively correlated with CSF glycine concentration (*r* = 0.415, *p* < 0.001) (Figure 1). CSF noradrenaline and GABA concentrations were not associated with labor pain score (*r* = −0.129, *p* = 0.2 and *r* = 0.024, *p* = 0.8, resp.). There were no significant correlations between pain duration and any of the neurotransmitter concentrations (Table 3). In the labor pain group, CSF glycine was significantly associated with CSF GABA (*r* = 0.51, *p* < 0.001), and CSF serotonin was significantly correlated with CSF glycine (*r* = 0.53, *p* < 0.001). There was no correlation between the other neurotransmitters (data not shown).

## 4. Discussion

This study demonstrated the effect of descending inhibitory control and spinal cord interneurons on pain modulation during active labor in an acute pain setting. Significant changes in CSF serotonin and glycine concentrations in pregnant women suffering from severe labor pain suggests the important role of serotonergic and glycinergic pathways in the central nervous system. Interestingly, there was an inverse correlation between CSF serotonin level and labor pain score (NRS). Additionally, we found CSF glycine level to be positively correlated with pain score, suggesting possible connection between the descending serotonergic pathway and glycinergic interneurons in the dorsal horn during visceral pain. However, the present study failed to demonstrate significant association between pain duration and the 4 studied neurotransmitters in cerebrospinal fluid.

### 4.1. Descending Inhibition (Noradrenaline, Serotonin)

Noradrenaline and serotonin released from the periaqueductal gray (PAG), the nucleus raphe magnus (NRM), and the locus coeruleus (LC) in the brain stem play a major role in descending control by modulating nociceptive signals in the dorsal horn prior to transmission of signals to the cortex [3]. In the present study, there was a significant decrease in CSF serotonin concentration in parturients with severe labor pain. Previous studies reported significant increase in CSF serotonin in pregnant women with painful active labor [visual analogue scale of 6.2 (IQR 4.8–7.6)]; however, the severity of pain was not intense [8]. It appears evident that the high level of pain intensity (NRS = 9) and the median pain duration of 325 minutes (5.4 hours) in the labor pain group in this study was sufficient to influence descending serotonergic control since an inverse correlation was observed between pain intensity and CSF serotonin level. Moreover, negative emotional effects, including depression and stress, were found to be related with lower CSF serotonin level [8]. However, anxiety and depression and their relation to lower CSF serotonin concentration were not evaluated in this study. Decreased serotonin level was also found in patients with long-standing severe pain caused by conditions that include trigeminal neuralgia, facial pain, and chronic headache [16]. Therefore, a lower level of serotonin in women enduring prolonged labor might reflect either pain or labor-induced stress.

It should be noted that this study showed no significant difference in CSF noradrenaline levels between the control and labor pain groups. In contrast, previous studies reported CSF noradrenaline level to be significantly decreased in patients with posttraumatic pain [8] and postsurgical pain [10], both of which are somatic pain. These findings make us question whether or not the descending noradrenergic pathways underlying the pain mechanisms of visceral pain and somatic pain are different. Further investigation is warranted to clarify and perhaps differentiate the role of the descending noradrenergic pathway in visceral and somatic pain.

### 4.2. Inhibitory Interneurons (GABA, Glycine)

According to the gate control theory, local interneurons in the dorsal horn release the inhibitory neurotransmitters GABA and glycine to modulate nociceptive impulse [2]. In the present study, the CSF glycine level in pregnant women with labor pain was significantly higher than in pregnant women without labor pain, suggesting the activation of glycinergic neurons in the spinal dorsal horn. A recent study revealed markedly elevated CSF glycine concentration in pregnant women with labor pain compared to those without labor pain [12]. Glycinergic neurons are recognized as inhibitory interneurons in the spinal dorsal horn synapsing on spinothalamic tract cells before transmitting modulating signal propagation to the supraspinal level [17]. However, the findings of this study do not support the antinociceptive role of GABA in the dorsal horn in the context of labor pain. We did, however, find a correlation between CSF glycine level and CSF GABA level in our analysis. The corelease of GABA and glycine in the spinal dorsal horn was observed in several animal studies [18–20], providing inhibitory postsynaptic currents (IPSCs) and attenuation of the spinothalamic tract signal. It appears that activation of glycinergic interneurons at the spinal dorsal horn is rapid and extensive, especially in acute pain. Thus, these findings emphasize the pivotal role of glycinergic inhibition in nociceptive modulation in pain mechanism and the possible link between glycine and GABA interneurons.

### 4.3. Link between Descending Inhibitory System and Inhibitory Interneurons

Most studies using animal models established a link between the descending inhibitory system and inhibitory interneurons in the spinal dorsal horn [6, 7]. Compelling evidence from electrophysiological study that focused on the spinal dorsal horn in rats showed that there were several subtypes of 5-HT receptors located in the dorsal horn and that their activation would potentiate inhibitory interneurons to release glycine [4, 5]. The present study is the first to demonstrate the correlation between CSF concentrations of serotonin and glycine in the labor pain group, suggesting a possible link between the serotonergic pathway and glycinergic interneurons during labor pain in pregnant women.

The main limitation of this study is that we did not separately evaluate each neurotransmitter receptor subtype, which is in contrast to studies conducted in animal model. CSF neurotransmitter concentrations in the pain pathway may be affected by stress or mood. In addition, our findings are limited by a relatively small study population and by our cross-sectional study design. As such, no conclusions regarding cause and effect relationships can be drawn. Moreover, the measurement of CSF neurotransmitters following lumbar puncture may not represent activity at only the spinal cord, as CSF flow is distributed throughout the entire central nervous system, including the cerebral cortex and the midbrain. However, CSF collected from the lumbar area is near the spinal cord. It may then be argued that lumbar CSF is enriched in and has a concentration of neurotransmitters that are produced in and that emanate from the spinal cord. Lastly, age and indications for cesarean section may be confounding factors that limit the present study, as there were significant differences between groups for both variables.

In conclusion, CSF serotonin and glycine were significantly correlated with labor pain scores. The findings of this study suggest that serotonergic and glycinergic systems may play a role in spinal modulation of visceral pain. In acute pain, the descending serotonergic system is inhibited, whereas glycinergic interneurons are activated. Statistically significant association was observed between glycine and GABA interneurons. The interrelationship between the descending serotonergic system and glycinergic interneurons needs to be further elucidated. Further studies are also needed to clarify the role of these inhibitory neurotransmitters in order to gain better understanding of neuronal mechanism in the pain pathway. The results of this study increase our collective understanding of the effect of these inhibitory neurotransmitters on pain modulation in the spinal dorsal horn.

## Figures and Tables

**Table 1:** Demographic, anthropometric, and clinical characteristics of pregnant women in the control and labor pain groups.

	Control group (*n* = 43)	Labor pain group (*n* = 45)	*p* value
Age (years), mean ± SD	32.81 ± 4.71	29.87 ± 5.52	0.008^*∗*^
Weight (kg), mean ± SD	71.15 ± 12.02	70.82 ± 12.22	0.9
Height (cm), mean ± SD	156.35 ± 5.17	157.56 ± 5.66	0.3
Body mass index (kg/m^2^), mean ± SD	29.05 ± 4.23	28.52 ± 4.64	0.58
Gestational age (weeks), mean ± SD	38.33 ± 0.89	38.36 ± 1.00	0.88
Indication for C/S, *n* (%)			0.001^*∗*^
(i) CPD	13 (30.22%)	31 (68.90%)	
(ii) Previous C/S	23 (53.49%)	10 (22.22%)	
(iii) Malrotation of fetus	5 (11.63%)	4 (8.88%)	
(iv) Twins	1 (2.33%)	0 (0%)	
(v) Previous surgery	1 (2.33%)	0 (0%)	
Preoperative pain score (NRS), median (IQR) (P25–P75)	0 (0-0)	9 (7–10)	
(i) Number of patients, NRS 5, *n* (%)	0 (0%)	0 (0%)	
(ii) Number of patients, NRS 6, *n* (%)	0 (0%)	8 (17.78%)	
(iii) Number of patients, NRS 7, *n* (%)	0 (0%)	4 (8.88%)	
(iv) Number of patients, NRS 8, *n* (%)	0 (0%)	9 (20.00%)	
(v) Number of patients, NRS 9, *n* (%)	0 (0%)	8 (17.78%)	
(vi) Number of patients, NRS 10, *n* (%)	0 (0%)	16 (35.56%)	
Pain duration (minutes), median (IQR) (P25–P75)	0 (0-0)	325 (240–525)	
Preoperative pain treatment, *n* (%)			
(i) No treatment	43 (100%)	28 (62.22%)	
(ii) Received pain medication	0 (0%)	17 (37.78%)	
Dose of pethidine administration, *n* (%)			
(i) 50 mg IM	0 (0%)	13 (28.89%)	
(ii) 75 mg IM	0 (0%)	4 (8.89%)	

^*∗*^ *p* value < 0.05 indicates statistical significance. CPD, cephalopelvic disproportion; C/S, cesarean section; IM, intramuscular; IQR, interquartile range; NRS, numerical rating scale; P, percentile; SD, standard deviation.

**Table 2:** Concentrations of CSF neurotransmitters in pregnant women in the control and labor pain groups.

CSF neurotransmitters	Control group (*n* = 43)	Labor pain (*n* = 45)	*p* value
Noradrenaline (ng/ml)	1.58 (1.03–1.91)	1.39 (1.13–1.62)	0.18
Serotonin (ng/ml)	5.44 (3.90–6.88)	3.76 (2.85–4.68)	<0.001^*∗*^
GABA (*μ*M)	0.04 (0.02–0.07)	0.03 (0.02–0.04)	0.08
Glycine (*μ*M)	19.13 (17.09–22.58)	25.22 (20.18–31.83)	<0.001^*∗*^

Data shown as median and interquartile range (P25–P75). ^*∗*^*p* value < 0.05 indicates statistical significance. GABA, gamma-aminobutyric acid.

**Table 3:** Correlation analysis between CSF neurotransmitters and pain duration.

	Noradrenaline	Serotonin	GABA	Glycine
Duration of pain				
*r*	−0.127	−0.068	−0.014	−0.126
*p* value	0.406	0.659	0.927	0.408

GABA, gamma-aminobutyric acid.
